# The role of prothrombin complex concentrates in reversal of target specific anticoagulants

**DOI:** 10.1186/1477-9560-12-8

**Published:** 2014-04-17

**Authors:** Katrina Babilonia, Toby Trujillo

**Affiliations:** 1 University of Colorado Hospital, Anschutz Inpatient Pavilion Tower 2, 12505 E 16th Ave, Mail Stop F 757, Aurora, CO 80045, USA; 2 University of Colorado Skaggs School of Pharmacy and Pharmaceutical Sciences, C238-V20 Pharmacy & Pharmaceutical Sciences, 12850 E. Montview Blvd. Room V20-1217, Aurora, CO 80045, USA

## Abstract

Over the past several years a new era for patients requiring anticoagulation has arrived. The approval of new target specific oral anticoagulants offers practitioners several advantages over traditionally used vitamin K antagonist agents including predictable pharmacokinetics, rapid onset of action, comparable efficacy and safety, all without the need for routine monitoring. Despite these benefits, hemorrhagic complicates are inevitable with any anticoagulation treatment. One of the major disadvantages of the new oral anticoagulants is lack of specific antidotes or reversal agents for patients with serious bleeding or need for urgent surgery. As use of the new target specific oral anticoagulants continues to increase, practitioners will need to understand both the pharmacodynamics and pharmacokinetic properties of the agents, as well as, the available literature with use of non-specific therapies to reverse anticoagulation. Four factor prothrombin complex concentrates have been available for several years in Europe, and recently became available in the United States with approval of Kcentra. These products have shown efficacy in reversing anticoagulation from vitamin K antagonists, however their usefulness with the new target specific oral anticoagulants is poorly understood. This article will review the properties of dabigatran, rivaroxaban and apixaban, as well as the limited literature available on the effectiveness of prothrombin complex concentrates in reversal of their anticoagulant effects. Additional studies are needed to more accurately define the role of prothrombin complex concentrates in patients with life threatening bleeding or who require emergent surgery, as current data is both limited and conflicting.

## Introduction

Anticoagulation is a routine intervention for the management of arterial and venous thromboembolic events across a wide variety of clinical situations. Despite the robust clinical history of anticoagulant development and clinical use, as recently as 2009 vitamin K antagonists (VKAs) had been the only oral anticoagulants available for the prevention and treatment of thrombosis. The availability of new target specific oral anticoagulants (TSOACs) has now changed that paradigm. The introduction of the direct thrombin inhibitor (DTI) dabigatran, as well as the factor Xa inhibitors rivaroxaban and apixaban represent potentially attractive alternatives to VKAs. The TSOACs offer several advantages over VKAs including predictable pharmacokinetics, rapid onset of action, and comparable efficacy and safety. The pharmacokinetic advantages allow for fixed dosing, and mitigate the need for routine laboratory monitoring or the need for bridging in the perioperative setting. A number of recent clinical trials have resulted in the Food and Drug Administration (FDA) approval of dabigatran, rivaroxaban and apixaban for stroke prevention in nonvalvular atrial fibrillation (AF) [1-3]. Rivaroxaban is also FDA approved for the prevention of venous thromboembolism (VTE) after orthopedic surgery, and very recently was approved for treatment of VTE [4]. The TSOACs also have been approved for a variety of indications by various accrediting bodies around the world (Table 1). There is ongoing research investigating the use of TSOACs for VTE prophylaxis in hospitalized medically-ill patients, and patients with acute coronary syndrome [5-7]. As the U.S. population ages, and research continues, it is likely that these TSOACs will be prescribed for more FDA approved, as well as off-label uses.

**Table 1:** Current approval of the NOACs

	**FDA Approval**	**EMA Approval**
**Dabigatran**	-Stroke prevention in AF	-VTE prevention after orthopedic surgery,
		-Stroke prevention in AF
**Rivaroxaban**	-Stroke prevention in AF,	-VTE prevention afte orthopedic surgery
	-VTE prevention after orthopedic surgery	-Stroke prevention in AF
	- VTE treatment	
**Apixaban**	-Stroke prevention in AF	-VTE prevention after orthopedic surgery
		-Stroke prevention in AF

EMA = European Medicines Agency, AF = Non- valvular Atrial Fibrillation, VTE = Venous Thromboembolism to include Deep vein thrombosis and Pulmonary Embolism.

Despite the many favorable attributes that TSOACs possess when compared to VKAs, they present unique clinical challenges of their own. As such, it is unlikely that TSOACs will replace VKAs in all patients. The paucity of information regarding certain clinical situations may present difficult challenges for clinicians in both the ambulatory and acute care environments. All of the TSOACs generally share similar rates of major bleeding when compared to VKAs, with specific agents showing reductions in bleeding rates for specific disease states [8]. Differences in types of bleeding may be observed though, with intracranial hemorrhage lower and GI bleeding generally higher as compared to VKAs. (1–3). However, bleeding risk is not zero, and management of patients who bleed while on a TSOAC is complicated by the lack of effective methods for laboratory monitoring, and emergent reversal of these medications remain unavailable or poorly understood. As prescribing and use of TSOACs increase, it will be increasingly important for clinicians to understand the pharmacokinetic/pharmacodynamic aspects of individual agents, as well as available evidence regarding the management of bleeding.

### Mechanism of action and pharmacokinetics

Dabigatran is a reversible inhibitor of factor IIa (thrombin) that binds directly to the active site on the thrombin molecule. Dabigatran is available as a prodrug, dabigatran etexilate, which is rapidly converted to the active drug dabigatran upon oral administration. Peak plasma concentrations occur within 1–3 hours, with a half-life of 12–14 hours in patients with normal kidney function [9]. Plasma concentration has been shown to correlate directly with anticoagulant effect [10]. Dabigatran is predominately excreted unchanged by the kidneys (80%). In patients with a creatinine clearance (CrCl) less than 30 ml/min the half-life increases to approximately 27 hours [11]. Dabigatran does not interact with the cytochrome P450 enzymes; however it is a substrate for p-glycoprotein and is not devoid of potential drug-drug interactions.

Rivaroxaban is a reversible inhibitor of both free and clot bound factor Xa. Upon oral ingestion it is rapidly absorbed with peak plasma concentrations occurring in approximately 2–4 hours. Plasma concentration correlates directly with anticoagulant effect. Renal function is important in elimination with one-third of the parent compound eliminated unchanged in the urine, one-third is eliminated in the urine as inactive metabolite, and the remaining one-third is eliminated in the feces [12]. Enough parent compound is cleared through the kidneys such that with CrCl greater than 80 ml/min the half-life of rivaroxaban is 8.3 hours, increasing to 9.5 hours in individuals with CrCl less than 30 ml/min [12]. Rivaroxaban does have significant liver metabolism, specifically through Cytochrome P450 3A4, and is also a substrate of p- glycoprotein. As such, potential drug-drug interactions must be accounted for. Apixaban is also a reversible inhibitor of both free and clot bound factor Xa. Peak plasma levels are achieved 1–3 hours after ingestion, and half-life is 10–14 hours in patients with normal renal function. Like dabigatran and rivaroxaban, plasma concentrations correlate directly with anticoagulant effect. Apixaban has similar characteristics to rivaroxaban in that 25% of the parent compound is cleared through the kidneys, it undergoes significant hepatic metabolism through cytochrome P450 3A4, and is a substrate for p-glycoprotein. Reduced renal function and drug-drug interactions also have the potential to alter the expected pharmacokinetic and pharmacodynamics response [13]. Table 2 compares the TSOACs and their pharmacokinetics.

**Table 2:** TSOAC Pharmacokinetics

	**Dabigatran**	**Rivaroxaban**	**Apixaban**
Target	Factor IIa	Factor Xa	Factor Xa
Dosage Form	capsule	tablet	tablet
Bioavailability	6%	60-80%	50-85%
Time to Peak	1-2 hours	2-4 hours	1-3 hours
Metabolism	Conjugation; No CYP involvement	Oxidation via CYP3A4	Oxidation via CYP3A4
Renal Excretion	80%	33%	25%
Substrate of p- glycoprotein?	Yes	Yes	Yes
FDA approved dosing for stroke prevention in a- fib	150 mg twice daily for	20 mg by mouth once daily for patients CrCL > 50 ml/min	5 mg by mouth twice daily
	patients CrCL > 30 ml/min	15 mg by mouth once daily	2.5 mg by mouth twice daily for patients with 2 or more of the following: Age > 80, weight < 60 kg or Serum Cr > 1.5
	75 mg by mouth twice daily for CrCL 15–30 ml/min	for patients with CrCL 15-50 ml/min	
FDA approved dosing for VTE prevention in hip	N/A	10 mg once daily for patients with CrCL > 30 ml/min	N/A
and knee			
replacement			
FDA approved	N/A	15 mg by mouth twice daily	N/A
dosing for {1) treatment of acute DVT or PE, or {2) long term prevention of		for 21 days, then 20 mg once daily for patients with CrCL > 30 ml/min	
recurrent DVTfPE		20 mg once daily for patients with CrCL > 30 ml/min	

CrCl = Creatinine Clearance, CYP = Cytochrome P450.

When prescribed appropriately, few patients should experience clinically significant bleeding while on TSOAC therapy. When it does occur however, clinicians need to have an appreciation for the mechanisms of action, clearance, and half-lives of agents to properly manage the situation. While the TSOACs generally produce a much more consistent dose–response as compared to oral VKAs, there are sources of potential pharmacokinetic variability that are important to consider when challenged with a patient who may be bleeding in the setting of TSOACs exposure. One drawback for the TSOACs is the lack of a readily available and reliable coagulation assay to provide quantitative information on the level of anticoagulation. As such, clinicians are required to make vital decisions with imprecise data. An in depth discussion of the impact of TSOACs on available coagulation assays is beyond the scope of this review and clinicians are referred to several recent reviews on this topic [14,15].

### Strategies for anticoagulation reversal

Patients presenting with anticoagulant-related bleeding while receiving TSOAC therapy should ideally be managed according to a pre-determined approach as determined by institutional guidelines. Details of this approach will likely be influenced by the pharmacology of the specific agent, the urgency of the clinical situation as well as the severity of bleeding. Options may include (1) observation and withholding anticoagulant therapy alone; (2) administering a specific reversal agent if one is available; (3) administration of supplemental clotting factors either via fresh frozen plasma (FFP) or prothrombin complex concentrates (PCCs); (4) Administration of prohemostatic agents such as activated prothrombin complex concentrates (aPCC or FEIBA) or recombinant factor VIIa (rFVIIa). Any of these interventions should always be preceded by appropriate supportive and symptomatic treatment, including mechanical compression or surgical intervention.

Due to their relatively short half-lives, supportive care and observation may be adequate for the majority of patients experiencing bleeding while on TSOAC therapy. Provided the patient has adequate renal function and/or no significant drug-drug interactions, it would be expected that clinically significant plasma concentrations of TSOAC therapy would be absent 24–36 hours after the last administered dose. Given each of the new TSOAC agents have no available antidote, there has been significant considerations in the use of either factor replacement therapy, or prohemostatic agents in the management of bleeding patients. However, as will be subsequently discussed there is little information to guide the clinician on the best approach with these agents. The presence of one of the potential factors listed in Table 3 may be helpful in recognizing whether bleeding may be due to potential over-anticoagulation, and whether aggressive interventions such as administration of a pro-hemostatic agent may be warranted.

**Table 3:** Metabolism of the TSOACs

	**Dabigatran**	**Rivaroxaban**	**Apixaban**
Renal Impairment	6 fold higher exposure when CrCL 10–30 ml/min	1.6 fold higher exposure when CrCL 15-29	1.44 fold higher exposure when CrCL 15-29
Age	Age > 75 = 30% increase in trough concentrations	Mean AUC 1.5 fold higher in age > 65	Mean AUC 1.3 fold higher in age > 65
Hepatic Impairment	N/A	2.3 fold increase exposure in Child-Pugh B	N/A
Drug-drug interactions	Avoid strong inhibitors or inducers of p-glycoprotein	Avoid strong inhibitors of p-glycoprotein and CYP 3A4	Avoid strong inhibitors of p-glycoprotein and CYP 3A4

AUC = Area under the curve CrCL = Creatinine Clearance CYP = Cytochrome P450.

### Prothrombin Complex Concentrates (PCC)

Prothrombin Complex Concentrates (PCC) are a concentrated plasma product that contains clotting factors in varying amounts. Formulations can include 3 factor products (II, IX and X) or 4 factor products (II, VII, IX and X). Dosing of PCC products is expressed as units of factor IX [16]. The factors provided by PCC products are generally not activated and require activation by the clotting cascade. There is one activated PCC (aPCC) product available, FEIBA, which contains activated Factor VII and inactivated forms of Factors II, IX and X [17]. Available inactive PCC products contain varying amounts of factors II, VII, IX and X. Some products may also contain coagulation inhibitors such as heparin, antithrombin, protein C, protein S and protein Z to mitigate thrombotic risk [18]. PCC formulations are listed in Table 4.

**Table 4:** **Prothrombin complex concentrates composition**^**a**^

**Prothrombin complex concentrate**	**Factor levels (IU/ml)**				**Protein levels ( IU/ml)**			**Other**	
	**II**	**VII**	**IX**	**X**	**C**	**S**	**Z**	**ATIII**	**Heparin**
**3 Factor**									
Bebulin	24-37	< 5	24-37	24-37	NA	NA	NO	None	< 0.15/IU FIX
Profilnine	NMT 150/U/100 Factor IX U	NMT 35/U/100 Factor IX U	100 unit	NMT 100/U/100 Factor IX U	NA	NA	NA	None	None
**4 Factor**									
Beriplex	20-48	10-25	20-31	22-60	22-31	17-19	Yes	Yes	Yes
Cofact	30	13	23	26	4	21	Yes	Yes	None
Kcentra	19-40	10-25	20-31	25-51	21-41	12-23	No	Yes	Yes
Octaplex	31	16	22	24	12	24	Yes	No	Yes
**Activated PCC**									
FEIBA*	1.3 IU/IU	0.9 IU/IU	1.4 IU/IU	1.1 IU/IU	1.1 IU/IU	NA	NA	No	No

^a^All concentrations are approximate and vary from one lot to another.

NMT = not more than, IU = international units.

*IU/IU = IU/FEIBA unit.

PCCs were originally created as a replacement product for Factor IX in Hemophilia B patients. These products are now largely used for emergency reversal of VKA therapy [19]. PCC dosing for anticoagulation reversal varies from 25–50 IU/kg. Several studies have demonstrated PCC efficacy in reversal of VKA anticoagulation for cases of emergent bleeding and emergent surgery [20-23]. Studies comparing effectiveness of PCC to fresh frozen plasma (FFP) indicate that PCC products provide a more rapid decrease in INR values as compared to FFP [20-23]. The 2012 CHEST Antithrombotic Guidelines recommend the use of PCC over FFP for patients with life-threatening bleeds while on VKA therapy [24]. However, this recommendation was Grade 2C, which indicates it is a weak recommendation with low quality evidence [24]. PCCs have also been evaluated and may be beneficial for off label use to restore hemostasis in excessive bleeding following surgery and major trauma [25,26].

While FFP and vitamin K have been the mainstay of VKA reversal and management of bleeding, these therapies are less likely to be efficacious in bleeding associated with TSAOCs. The goal of anticoagulation reversal with TSAOCs is the overwhelm inhibition of direct thrombin or factor Xa. FFP contains much lower amounts of clotting factors II, VII, IX and X when compared to PCC products. Therefore, the amount of FFP required to administer an equivalent factor replacement dose received in PCC products would be 8–16 units of FFP [27]. The amount of FFP and volume required to administer the reversal dose would likely not be feasible in urgent bleeding scenarios. In addition, patients with cardiac, pulmonary and renal issues may not be able to tolerate such large amounts of volume. PCC products may offer a number of additional advantages over FFP. FFP is frequently available only via the hospital blood bank and must be thawed prior to administration. The time involved with procurement and administration of agent may be problematic. PCC products go through at least one viral inactivation step to reduce the risk of viral pathogen transmission [28]. Finally, unlike FFP, PCC’s are free of leukocytes, so they are less likely to cause transfusion related acute lung injury (TRALI) or infusion reactions [28]. PCC product labeling does include a warning of potential arterial and venous thrombotic complications associated with its use [29]. Current PCC formulations differ greatly from preparations used in the 1970-80’s, and generally are thought to have a lower thrombosis risk [28]. For example, many of today’s PCC formulations contain coagulation inhibitors such as heparin, antithrombin, protein C, protein S and protein Z which allows for more balanced replacement of procoagulants factors and anticoagulants proteins [28]. Evidence suggests that the primary determinant of the thrombotic risk associated with PCC use is accumulation of factor II, which is associated with large or frequent dosing [28]. PCC use in patients who are predisposed to thrombotic complications (i.e. patients who require outpatient anticoagulation therapy) makes it difficult to identify if thrombotic events are attributable to PCC use or to patient comorbidities [28]. Therefore, practitioners considering anticoagulation reversal therapies should weigh the risk of thrombotic complications from treatment with PCC against the need for rapid correction of coagulopathy.

### Dabigatran reversal

#### ***Animal studies***

In an in-vivo animal study, rats received high doses of dabigatran (30 mg/kg) or placebo via gastric lavage. The reversal effects of PCC (Beriplex, Octaplex), activated recombinant Factor VII (rVIIa) (Novoseven) and activated PCC (FEIBA) were evaluated using tail bleeding times and coagulation parameters including thrombin time (TT), activated partial thromboplastin time (aPTT), prothrobin time (PT) and ecarin clotting time (ECT). Bleeding times were increased from 171 sec in control subjects to 495 sec in subjects receiving dabigatran. All coagulation labs were prolonged 3–5 fold baseline following dabigatran administration. Administration of 35 IU/kg Beriplex, 40 IU/kg Octaplex, 0.5 mcg/kg rVIIa or 100 IU/kg FEIBA completely reversed dabigatran associated bleeding times to baseline within 5 min of IV administration. This effect was maintained for the 2 hour study period. Despite the effects on bleeding time, coagulation parameters including TT, aPTT and ECT remained prolonged after administration of the PCC products. Only PT was reversed to baseline levels [30].

Another in vivo rat study evaluated rFVIIa (Novoseven) or activated PCC (FEIBA) on bleeding times and aPTT following administration of supratherapeutic doses of dabigatran. Bleeding times were significantly increased with dabigatran compared to controls, with 1455 sec vs 145 sec. Administration of rFVIIa (0.1 or 0.5 mcg/kg) or FEIBA (50 u/kg or 100 u/kg) significantly decreased bleeding times to 186 sec and 135 sec with rFVIIa, or 146 sec and 174 sec with FEIBA. Effects on coagulation assays were noted again to be discordant with effects on bleeding. aPTT was dose dependently decreased by rFVIIa but not by FEIBA [31].

An in vivo rabbit study evaluated the effect of 4-Factor PCC (Beriplex) in rabbits treated with dabigatran who then underwent kidney incision. Subjects received dabigatran 0.4 mg/kg, followed by administration of saline or PCC with doses of 20 u/kg, 35 u/kg or 50 u/kg. Blood loss in control subjects ranged from 1–7.2 ml. Administration of dabigatran increased bleeding to 15–46 ml. Blood loss was significantly diminished with increasing PCC doses, with a decrease in bleeding volume of 5.44 ml per 10 IU/kg increment increase in PCC dose. The 50 IU/kg PCC dose decreased blood loss to volumes similar to that seen in the control subjects. Time to hemostasis was 20 min in patients treated with dabigatran followed by saline. This time was also decreased with increasing doses of PCC, falling to a median of 5.7 min in the highest PCC dose (50 u/kg) group [32].

### Human studies

In an ex vivo study, 10 healthy subjects received a single dose of dabigatran 150 mg. Blood samples were collected just prior to drug administration and then 2 hours after to assess peak concentrations. Reversal agents administered included 4-Factor PCC (Kanokad), rFVIIa (Novoseven), or activated PCC (FEIBA). Addition of 4-Factor PCC (25 u/kg) and FEIBA (80 u/kg) to blood samples resulted in a large increase in thrombin generation to above baseline levels. However, increased thrombin generation was not seen with rFVII [33].

Another study evaluated the effect of 4-Factor PCC (Cofact) on 12 healthy adults who had been administered dabigatran 150 BID for 2 and 1/2 days. The patients had baseline coagulation labs including aPTT, ECT and TT drawn, then received PCC (50 u/kg). Coagulation labs were collected and assessed at various intervals over the next 24 hours. PCC had no effect on coagulation assays at any interval of the study period [34].

### Case reports with excessive response to dabigatran

A 72 y/o female was started on dabigatran 220 mg daily for prophylaxis following total hip replacement (THR). Two days later, the patient developed dyspnea, hypotension, tachycardia and anemia. She received 2 units of packed red blood cells (PRBC’s) and was thought to have a pulmonary emboli (PE). Dabigatran was discontinued and the patient was started on enoxaparin 0.7 mg/kg BID. PE was later ruled out and enoxaparin was discontinued. The patients clinical status continued to worsen with hypovolemic shock and acute renal failure. A laparotomy was performed which revealed ischemic lesions requiring bowel resection. Prior to surgery (2 days after discontinuation of dabigatran, 1 day after discontinuation of enoxaparin) PT > 60 sec and aPTT > 150 sec. The patient received 22 u PRBC, 26 u FFP, 40 u/kg of PCC (Kaskadil), 66 mcg/kg of rFVIIa (Novoseven), 1.5 gm of figrinogen concentrate (Clottagen) and transexamic acid. Despite these measures, the patients PT and aPTT remained elevated. The patient died 2 hours after surgery [35].

A 67 year old male taking dabigatran 150 mg BID for atrial fibrillation experienced a life threatening bleed during a cardiac ablation procedure. The patient received his last dose of dabigatran 8 hours prior to the procedure. A trans-septal perforation occurred resulting in massive blood losses that exceeded 3 L via the pericardial window. The patient received FFP, protamine, and packed red blood cells with no reduction in bleeding. A low dose (26 u/kg) of activated PCC (FEIBA) was infused over 15 min. Signs of hemostasis were observed within minutes of initiation of the infusion. Bleeding had stopped by the completion of the PCC infusion [36].

### Rivaroxaban reversal

#### ***Animal studies***

In a rabbit study, subjects were randomized into 4 groups; control, rivaroxaban + saline, rivaroxaban + rFVIIa 150 mcg/kg, or rivaroxaban + PCC 40 u/kg (Kaskadil). Hepatosplenic blood loss was significantly increased in the rivaroxaban group as compared to controls, 17 gm vs 7 gm respectively. Neither rFVIIa or PCC reduced blood loss, 15 gm vs 19.5 gm, respectively. Additionally, laboratory coagulation tests, such as PT and aPTT, were increased significantly in the rivaroxaban treated group compared to controls. Recombinant FVIIa and PCC normalized aPTT and only partially corrected the PT. Neither reduced blood loss [37].

A rat experiment evaluated mesenteric bleeding times in subjects that had received rivaroxaban. Subjects were randomized to receive inactive PCC (25 u/kg, 50 u/kg), activated PCC (aPCC) (50 u/kg, 100 u/kg) or rFVIIa (100 mcg/kg, 400 mcg/kg). Administration of inactive PCC 50 u/kg significantly reduced bleeding times, however bleeding times with the 25 u/kg dose were similar to controls. Activated PCC reduced bleeding times at both doses. There was not an increased effect on bleeding times associated with use of the higher 100 u/kg aPCC dose. Finally, rFVIIa significantly reduced bleeding times at 400 mcg/kg dose. The 100 mcg/kg dose also decreased bleeding time, but it was not statistically significant [38].

In a baboon study, subjects received intravenous infusion of rivaroxaban, followed by an infusion of either aPCC 50 u/kg (FEIBA) or 210 mcg/kg rFVIIa (Novoseven). Upon completion of the aPCC infusion bleeding times had normalized. However 20 min after completion of the infusion, bleeding time increased to 1.7 times normal. In subjects treated with rFVIIa a 34% reduction in bleeding times were observed, but this did not reach statistical significance [38].

### Human studies

In the above mentioned ex vivo human study, the patients underwent a 2 week wash out period and were then administered 20 mg of rivaroxaban in a single dose. PCC (Kanokad), aPCC (FEIBA) or rFVIIa (Novoseven) were once again added to blood samples and evaluated ex vivo. In this case, PCC and rFVIIa only partially increased peak thrombin generation in samples. Activated PCC normalized thrombin generation [33].

Rivaroxaban was also evaluated in the previously discussed trial in which 12 healthy adults received therapeutic doses of rivaroxaban for 2 and ½ days, followed by administration of 50 u/kg of PCC (Cofact). In this case, PT was normalized almost immediately. These effects were sustained for 24 hours following administration [34].

### Apixaban reversal

#### ***Animal studies***

An in vivo study evaluated the effect of rFVII (Novoseven), PCC (Kanokad) and fibrinogen (Clottafact) in rabbits administered apixaban [39]. Subjects were given apixaban 0.4 mg/kg bolus, with 0.6 mg/kg/hr infusion then randomized to receive, rFVII 240 mcg/kg, PCC 60 IU/kg, or fibrinogen 300 mg/kg. Apixaban significantly increase hepatosplenic blood loss and bleeding times compared to control, 11.6 gm and 126.4 sec with apixaban vs 8.3 gm and 70.8 sec with control. Administration of either PCC or rFVII did not decrease hepatosplenic blood loss (11.8 gm and 12.2 respectively.) However, both PCC and rFVII were found to partially reverse bleeding time to 101.1 sec and 83.5 sec, respectively. Fibrinogen appeared to increase both blood loss and bleeding time in apixaban treated rabbits to 19.2 gm and 154.2 sec. However, paradoxically fibrinogen improved clot firmness and increased endogenous thrombin potential (ETP). The authors proposed that this paradoxical effect may be attributed to the numerous effects fibrin can exert on thrombin. Therefore, thrombin activity may not reflect it’s true coagulant potential. Apixaban modestly increased PT, but had no effect on aPTT. Only rFVII normalized prolonged PT values, PCC and fibrinogen had no effect [39].

### Human studies

There were no studies found that evaluated reversal strategies for apixaban anticoagulation in humans.

## Discussion

Patients with over anticoagulation or bleeding secondary to decreased levels of circulating clotting factors (as seen with VKA therapy) respond to factor replacement with FFP, PCC and vitamin K. Unfortunately, these strategies are less efficacious in patients receiving target specific inhibition of clotting factors as seen with direct thrombin and Factor Xa inhibitors. In these cases, the goal of reversal is not only to replace clotting factors, but to overwhelm the direct inhibition of factors in the latter phases of the coagulation cascade [27].

The pharmacokinetic profiles of the TSOACs differs greatly from that seen with VKA’s. Elimination of dabigatran, and to a lesser extent rivoraxaban and apixaban, is predominately renal [40-42]. This can be advantageous in patients with preserved renal function experiencing minor bleeding or requiring surgery, as a strategy of holding doses or delaying procedures may be sufficient. In addition, removal of dabigatran via hemodialysis may also provide benefit in patients with decreased renal clearance of the agent [40]. Unfortunately, cessation of the anticoagulant agent or initiation of hemodialysis may not be sufficient or feasible in patients with major bleeding or requiring emergent surgery.

One major disadvantages of the TSAOCs is the lack of specific antidotes for patients with excessive anticoagulation, major bleeding, or need for emergent surgery. Specific antidotes are in development for both the oral direct thrombin inhibitors and factor Xa inhibitors. A monoclonal antibody that neutralizes dabigatran is in clinical trials. Initial in vitro and ex vivo studies have demonstrated complete inhibition of dabigatran anticoagulant activity [43]. A reversal product is also in development for factor Xa inhibitor anticoagulants [44,45]. This product contains an inactive factor Xa molecule that can bind to factor Xa inhibitors (such as rivaroxaban or apixaban) and decrease their anticoagulant effect [44,45]. Currently, the product is entering into Phase II clinical trials [45].

The lack of reliable monitoring assays further complicates scenarios in which urgent anticoagulation reversal is required. The TSAOCs will prolong traditionally used coagulation assays, such as PT and aPTT, however sensitivity can vary greatly based on agent or assay manufacturer and lot [46,47]. In addition, normalization of coagulation assays in animal models did not always correlate with improvement in bleeding [27,38].

## Conclusions

### Dabigatran

Hemodialysis should be considered in patients with impaired renal function who require reversal of dabigatran for major bleeding or surgical procedures when available. PCC use decreased bleeding times in both rats and rabbits who had received dabigatran [30,32]. The available studies in human did not evaluate bleeding times, but instead focused on coagulation assays (aPTT, ECT, TT) or measures of thrombin generation [34,33]. These studies demonstrated that PCC was able to significantly increase thrombin generation in in vitro blood samples containing dabigatran, but did not correct coagulation assays commonly used to assess level of anticoagulation in vivo [34,33]. Activated PCC (FEIBA) corrected bleeding times in two rat models and proved to significantly increase thrombin generation in vitro in humans [30,31,33]. Based on these results, use of 4-factor PCC or aPCC would be reasonable choices for urgent reversal of patients with dabigatran associated major bleeding or need for emergent surgery. Activated PCC may carry a higher risk of thrombosis, and therefore may be considered as an alternative if a 4-factor PCC is not available.

### Rivaroxaban

PCC use did not decrease hepatosplenic bleeding in rabbits treated with rivaroxaban, but at high doses (50 u/kg) was able to decrease mesenteric bleeding in rats [37,38]. In human studies, PCC completely normalized both PT and endogenous thrombin potential (ETP) immediately following infusion, however it only partially increased peak thrombin generation in an ex vivo human model [34,33]. Activated PCC significantly improved bleeding times in rats and baboons treated with rivaroxaban, but in the baboon model the bleeding correction was not sustained [38]. In the human ex vivo evaluation aPCC normalized thrombin generation [33]. Based on this available evidence, use of either a 4-factor PCC or aPCC would be reasonable choices for reversal of rivaroxaban. The evidence supporting aPCC may be slightly more favorable, but consideration of the increased thrombosis risk for activated factor products must also be considered.

### Apixaban

PCC use did not decrease bleeding volumes, but was able to partially decrease bleeding times in rabbits. PCC was also not able to normalize elevated PTs associated with apixaban administration [39]. Despite the limited amount of data with apixaban reversal, it may be rational to apply the same reversal strategies used for rivaroxaban to apixaban based on their similar mechanism of action.

Additional studies are needed to evaluate the effectiveness of factor replacement for reversal of oral anticoagulants and the risk of thrombosis associated with these therapies.

## Competing interests

KB has no competing financial interests to disclose. TT is a consultant for the following; Janssen Pharmaceuticals, Boehringer-Ingelheim Pharmaceuticals, Pfizer/Bristol Myers-Squibb and Daichii-Sankyo.

## Authors’ contributions

KB and TT drafted and revised the manuscript, “The Role of Prothrombin Complex Concentrates in Reversal of Target Specific Anticoagulants”. Both authors contributed to this manuscript. Both authors read and approved the final manuscript.
